# Biological tapering is cost-effective in patients with inflammatory arthritis in the biosimilar era: secondary analyses on the randomised BIODOPT trial

**DOI:** 10.1016/j.ero.2025.07.002

**Published:** 2025-08-08

**Authors:** Line Uhrenholt, Mads Engell Refstrup Sørensen, Jan Sørensen, Anne Estrup Olesen, Robin Christensen, Lene Dreyer, Bente Glintborg, Ellen-Margrethe Hauge, Anne Gitte Loft, Mads Nyhuus Bendix Rasch, Hans Christian Horn, Annette Schlemmer, Salome Kristensen

**Affiliations:** 1 Center of Rheumatic Research Aalborg (CERRA), Department of Rheumatology, Aalborg University Hospital, Aalborg, Denmark; 2 Department of Clinical Medicine, Aalborg University, Aalborg, Denmark; 3 Danish Center for Clinical Health Services Research, Aalborg University, Aalborg, Denmark; 4 School of Population Health, RCSI University of Medicine and Health Sciences, Dublin, Ireland; 5 Department of Clinical Pharmacology, Aalborg University Hospital, Aalborg, Denmark; 6 Section for Biostatistics and Evidence-Based Research, Bispebjerg and Frederiksberg Hospital, the Parker Institute, Frederiksberg, Copenhagen, Denmark; 7 Research Unit of Rheumatology, Department of Clinical Research, University of Southern Denmark, Odense University Hospital, Odense, Denmark; 8 DANBIO and Copenhagen Center for Arthritis Research (COPECARE), Center for Rheumatology and Spine Diseases, Centre of Head and Orthopedics, Copenhagen University Hospital, Glostrup, Denmark; 9 Department of Clinical Medicine, Faculty of Health and Medical Sciences, University of Copenhagen, Copenhagen, Denmark; 10 Department of Rheumatology, Aarhus University Hospital, Aarhus, Denmark; 11 Department of Clinical Medicine, Aarhus University, Aarhus, Denmark; 12 Department of Rheumatology, Odense University Hospital, Odense, Denmark; 13 Department of Rheumatology, Randers Regional Hospital, Randers, Denmark

## Abstract

**Objectives:**

We assessed the cost-effectiveness of disease activity–guided tapering to continuation of biologics as usual care in inflammatory arthritis.

**Methods:**

Adults with rheumatoid arthritis, psoriatic arthritis, or axial spondyloarthritis on stable biological dose (including biosimilars) and in low disease activity ≥12 months were randomised (2:1) to disease activity–guided tapering or continuation of biologics as usual care.

The cost-effectiveness evaluation assessed the 18-month quality-adjusted life years (QALYs), hospital costs, and patient costs. Analyses were based on intention-to-treat. Missing short form six-dimensional (SF-6D) values were imputed with the mean SF-6D value in the relevant group at the specific time point. Patients lost to follow-up were assumed to continue their baseline biological dose onwards. We used a 2-sample *t* tests with unequal variance to capture between-groups differences with 95% CI.

**Results:**

Of 142 included patients, 78% were treated with biosimilars. At 18 months, 75% (71/95) of the tapering group and 17% (8/47) of the control group were on a tapered biological dose. We found no difference between groups in QALYs, mean difference: 0.008 points (95% CI: −0.008 to 0.024). Patients in the tapering group incurred significantly lower mean hospital costs than the control group, between-group difference: −1936€ (95% CI: −3753€ to −119€). Mean patient costs were comparable between groups: −52€ (95% CI: −350€ to 246€).

**Conclusions:**

Disease activity–guided tapering of biologics in a biosimilar era resulted in substantial reductions in 18-month hospital costs, whereas the impact on QALY and patient costs was similar. Thus, the tapering algorithm was cost-saving.

**WHAT IS ALREADY KNOWN ON THIS TOPIC**

• Increasing evidence supports that biological therapies can be gradually tapered in patients with RA, PsA, or axSpA in sustained low disease activity without risking loss of therapeutic response.
• In RA, tapering of originator biologics compared to continued treatment has been shown to be cost-saving with no or only minor differences in quality-adjusted life years.

**WHAT THIS STUDY ADDS**

• This is the first cost-effectiveness analysis to compare disease activity-guided tapering with continuation of biologics as usual care across RA, PsA, and axSpA using data on various biologics, including biosimilars.
• Tapering was found to be cost-effective, as a significant reduction in hospital cost was demonstrated without compromising quality-adjusted life years or increasing patient costs.
• A statistically significant and clinically relevant cost-saving was also observed among patients treated with biosimilars, emphasising that tapering remains relevant in the biosimilar era.

**HOW THIS STUDY MIGHT AFFECT RESEARCH, PRACTICE OR POLICY**

• The study confirms that disease activity-guided tapering of biologics, including biosimilars, is cost-effective in patients with RA, PsA, or axSpA supporting implementation in clinical practice.
• The findings may influence clinical guidelines and healthcare policies.

Alt-text: Unlabelled box

## INTRODUCTION

Biological therapies are widely used in the treatment of patients with rheumatoid arthritis (RA), psoriatic arthritis (PsA), and axial spondyloarthritis (axSpA). Traditionally, standard doses of biologics are maintained for life once patients achieve sustained remission or low disease activity (LDA). However, increasing evidence supports the possibility of gradually tapering biologics after a disease activity–guided algorithm while maintaining stable LDA [[1], [2], [3], [4], [5], [6], [7], [8], [9]]. Although an increased risk of arthritis flare has been reported when biologics have been tapered, dose escalation most often results in disease control with a low risk of persistent flare [10].

A recent survey among patients with well-controlled RA reported found that 63% were willing to consider biological tapering compared with only 36% of their physicians [11]. Previously, patient satisfaction and quality of life have been shown to improve with reduced drug dosages [12]. Therefore, a decrease in disease burden could be expected if LDA is maintained, as tapering implies less frequent biological injections and fewer outpatient visits for biological infusions. Furthermore, biological tapering is expected to reduce the risk of adverse drug reactions, including serious infections [13], and to reduce health care costs as treatment with biologics is expensive.

A few cost-effectiveness assessments have been conducted on biological tapering strategies based on clinical trial data in patients with RA treated with tumour-necrosis factor inhibitors (TNFis) [[14], [15], [16], [17]]. The majority reported decreased biological costs with no significant loss in quality-adjusted life years (QALYs) [[14], [15], [16]]. However, the STRASS trial observed a small but statistically significant decrease in QALY in the tapering group compared with the control group [17]; nonetheless, tapering still seemed cost-effective although the acceptability of QALY loss to cost savings was unclear. These cost-effectiveness analyses only included originator biological drugs [[14], [15], [16], [17]], but as biosimilars emerge, cost savings are expected to be markedly lower [[18], [19], [20], [21]] and might not outweigh potential costs related to tapering, eg, additional visits due to flare symptoms or biological switch due to persistent flare. Therefore, a cost-effectiveness evaluation of a tapering algorithm that includes biosimilars is needed.

The BIODOPT (BIOlogical Dose OPTimisation) trial recently evaluated disease activity–guided tapering of biologics to continuation of biologics as usual care in patients with RA, PsA, or axSpA in sustained LDA [6]. Overall, 78% of patients were treated with biosimilars during follow-up. At 18 months, one-third of the tapering group achieved ≥50% biologic dose reduction compared with the baseline dose. Disease activity remained similar between groups, as flares had a short duration due to quick dose escalation of the biological therapy. This secondary analysis on the BIODOPT trial aims to evaluate the cost-effectiveness of disease activity–guided tapering of biologics in comparison with continuation of biologics as usual care.

## METHODS

### Study design and participants

The BIODOPT study was conducted between May 2018 and September 2021 as a pragmatic, multicentre, randomised, open-label, equivalence trial [6,22]. Approval was obtained by the Danish Medicine Agency (2017091722), the North Denmark Region Committee on Health Research Ethics (N-20170073), and the Danish Data Protection Agency (2017-194). The study complied with the Declaration of Helsinki and was registered at EudraCT (2017-001970-41, December 21, 2017). All participants gave written informed consent before enrolment. Adults with a diagnosis of RA, PsA, or axSpA on stable biological dose and in LDA ≥12 months were eligible for inclusion. Patients with RA and PsA were monitored by Disease Activity Score28-C-Reactive Protein (DAS28-CRP) with LDA defined as a score ≤3.2 [23], and patients with axSpA were monitored by Ankylosing Spondylitis Disease Activity Score (ASDAS) with LDA defined as a score <2.1 [24]. After enrolment, patients were randomised (2:1) to tapering or control. The tapering group followed a disease activity–guided tapering algorithm with prolongation of the dosing interval by approximately 25% every 4 months until flare or biologic withdrawal. For infliximab, the dosing interval was spaced with 2 weeks at each infusion as described previously [22]. The control group was kept on their baseline biological dose; however, a small increase in the dosing interval was allowed (as usual practice) if requested by the patient.

In accordance with the Danish national recommendations, a mandatory switch from originator biologics to biosimilars was implemented in 2015 for infliximab [18], in 2016 for etanercept [19], and in 2018 for adalimumab [20]. A few patients remained on originator biologics despite the mandatory switch but were switched to the corresponding biosimilar between 2018 and 2020, ie, during the study period. Biological therapies are free of charge in Denmark; therefore, patients do not pay for their biological treatment.

### Effectiveness and cost assessment

The cost-effectiveness analysis included an assessment of QALY, hospital costs, and patient costs.

QALYs were calculated as the area under the curve of the short form six-dimension (SF-6D) index [25], based on data from the short form 36 questionnaire, which was collected at every trial visit, ie, baseline and months 4, 8, 12, and 18.

Hospital costs included biological treatment costs and extra outpatient visit costs due to possible symptoms of flare. Protocolised outpatient visits were similar for the tapering group and the control group, ie, baseline and months 4, 8, 12, and 18, and therefore not included in the analyses. Information on extra visits to other departments, the general practitioner, the physical therapist, hospitalisation, loss of productivity, or sick leave were not obtained and therefore not included in the analyses.

The biological treatment costs comprised biological drug costs, costs related to the switch of biological therapy, and biologic administration costs, including salary for the use of nurses and costs related to infusion of biological therapies. Based on expert opinion, infusion seat time was prespecified to 120 minutes per patient per intravenous biologic, with 60 minutes dedicated to nurse time. Handout of subcutaneous biologics in the outpatient clinic was prespecified to require 15 minutes of nurse and patient time per contact. Based on data from the Danish Medicines Council, hourly costs for nurses were 59.27€ [26], and hourly costs related to infusion of intravenous biologics were 5.43€ [27]. Data on individual patient’s administration of biological drugs were registered throughout the study in the electronic case report form in Research Electronic Data Capture [28,29]. Biological costs were obtained from Aalborg University Hospital Pharmacy’s database; the actual hospital purchase prices in 2022, including negotiated discounts, were used in the analyses. The biosimilar prices for adalimumab, etanercept, and infliximab were applied in the analyses, as patients on these agents were switched to the biosimilar product in accordance with the national recommendations. The World Health Organisation’s defined daily doses were applied for the analyses. Extra outpatient visits were predefined to require 45 minutes of physician and patient time. Based on data from the Danish Medicines Council, hourly costs for a chief physician were 137.63€ [26].

Patient costs included patient transport costs and patient time costs. Transport costs were based on the distance from the individual patient’s home address to the hospital and back, obtained from Google Maps. Based on data from the Danish Medicines Council, patient transport costs were, in accordance with the Danish transportation rate, 0.47€/km [26]. Patient time included transportation time throughout the study period, time related to receive biologics, and time related to extra visits. Based on data from the Danish Medicines Council, patient time costs were, in accordance with reports from Statistics Denmark, 24.33€ per hour [26].

### Statistical analysis

Data from the BIODOPT trial were analysed in accordance with a prespecified statistical and health economic analysis plan and the recommendations of the EQUATOR network [30], ie, the CONSORT and CHEERS statements [[31], [32], [33]]. Intention-to-treat analyses were applied, ie, all randomised participants with available baseline data were included independently of subsequent protocol deviations [34].

In STATA, the SF-6D index scores at each trial visit were connected with straight lines to calculate the area under the SF-6D index curve, which corresponds to QALY at 18 months. Missing SF-6D values were handled by ‘single-step’ mean imputation. Specifically, missing values were imputed with the mean SF-6D score at the corresponding time point within the same randomised group (ie, the tapering group if the patient belonged to the tapering group or the control group if the patient belonged to the control group). A sensitivity analysis based on the last observation carried forward was performed to assess the implication of missing SF-6D values. Patients lost to follow-up for any reason were assumed to have continued their baseline biological dose after loss to follow-up. Differences between groups are presented with 95% CI and analysed using 2-sample *t* tests with unequal variance. Bootstrapped CIs were estimated and provided similar results to the parametric confidence intervals and were therefore not reported. Costs are reported in 2022 Euro (€) and time costs in hours. Subgroup analyses on hospital costs at 18 months stratified by LDA, diagnosis (RA, PsA, or axSpA), and biological mode of action (TNFi or non-TNFi) were conducted to explore potential differences. All analyses were performed using STATA version 18.

## RESULTS

As presented previously, 142 patients were included in the BIODOPT trial, with 95 patients allocated to tapering and 47 patients allocated to control [6]. Loss to follow-up was 6% (8/142). Baseline characteristics, including the SF-6D index, were well-balanced as presented in Table 1. The majority of patients (78% [111/142]) were treated with adalimumab, etanercept, or infliximab and thus had been switched to the corresponding biosimilar in accordance with national recommendations. As specified in the Methods section, the biosimilar prices for adalimumab, etanercept, and infliximab were applied for these analyses.

**Table 1:** Baseline characteristics in the intention-to-treat population

Variable	Tapering group *N* = 95	Control group *N* = 47
Women, *n* (%)	52 (55%)	20 (43%)
Age (y), mean (SD)	51.9 (15.4)	52.3 (15.9)
Diagnosis:		
RA, *n* (%)	41 (43%)	20 (43%)
PsA, *n* (%)	18 (19%)	8 (17%)
AxSpA, *n* (%)	36 (38%)	19 (40%)
Disease duration (y), median (IQR)	11 (6-18)	12 (6–20)
On csDMARDs, *n* (%)	41 (43%)	22 (47%)
Baseline biological therapy:		
Abatacept, *n* (%)	1 (1%)	3 (6%)
Adalimumab, *n* (%)	17 (18%)	10 (22%)
Certolizumab-pegol, *n* (%)	8 (9%)	1 (2%)
Etanercept, *n* (%)	20 (21%)	6 (13%)
Golimumab, *n* (%)	6 (6%)	3 (6%)
Infliximab, *n* (%)	37 (39%)	21 (45%)
Tocilizumab, *n* (%)	6 (6%)	3 (6%)
Duration of baseline biologic (years), median (IQR)	5 (2–8)	4 (3–11)
Repeated biological failure^a^, *n* (%)	6 (6%)	3 (6%)
PASS ‘yes’, *n* (%)	90 (95%)	44 (94%)
SF-36 PCS (0-100), median (IQR)	52.0 (47.4–55.1)	49.8 (44.2–52.6)^b^
SF-36 MCS (0-100), median (IQR)	56.4 (47.3–59.5)	54.5 (48.5–59.6)^b^
SF-6D index (0-1), median (IQR)	0.82 (0.81–0.82)	0.82 (0.80–0.84)^b^
Tender joint count (0-68), median (IQR)	0 (0–0)	0 (0–0)
Swollen joint count (0-66), median (IQR)	0 (0–0)	0 (0–0)
CRP (mg/L), median (IQR)	2.6 (0.8–3.9)	2.2 (0.6–3.9)
In remission^c^, *n* (%)	82 (86%)	40 (85%)

N, number; SD, standard deviation; RA, rheumatoid arthritis; PsA, psoriatic arthritis; axSpA, axial spondyloarthritis; IQR, interquartile range; csDMARDs, conventional synthetic disease-modifying antirheumatic drugs; PASS, patient acceptable symptom state; SF-36, short form health survey 36; PCS, physical component summary; MCS, mental component summary; SF-6D, short form six-dimensional health survey; CRP, C-reactive protein; mg, milligramme; L, litre.

a Patients on biological agent number ≥3.

b One missing value (SF-36 not performed at baseline).

c Evaluated as Disease Activity Score (DAS)28-CRP <2.6 for RA and PsA and Ankylosing Spondylitis Disease Activity Score (ASDAS) <1.3 for axSpA.

### SF-6D index and QALYs

**Figure 1:**
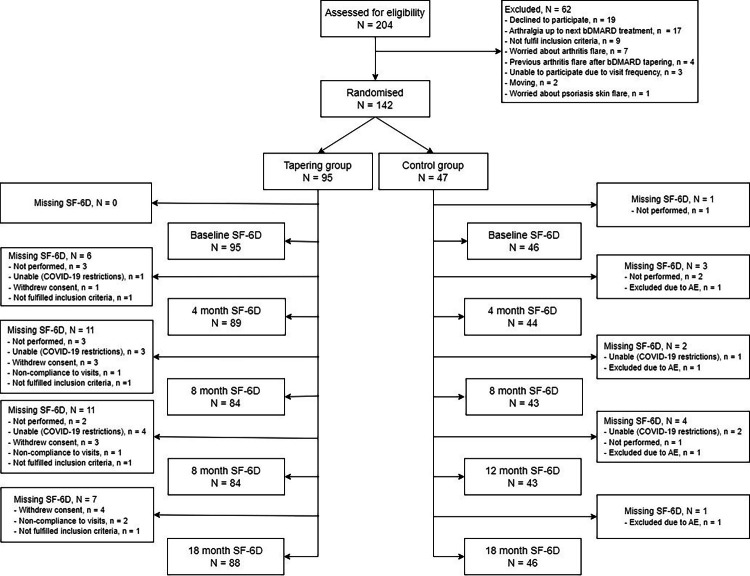
Flow diagram of the study population including assessment of SF-6D. SF-6D, short form six-dimensional.

Missing values of the SF-6D index during the study period were limited, as shown in Figure 1. The majority of missing values were due to study discontinuation; however, some were due to implications of the COVID-19 pandemic, ie, telephone consultation due to lockdown during which the SF-36 paper form was not completed.

**Figure 2:**
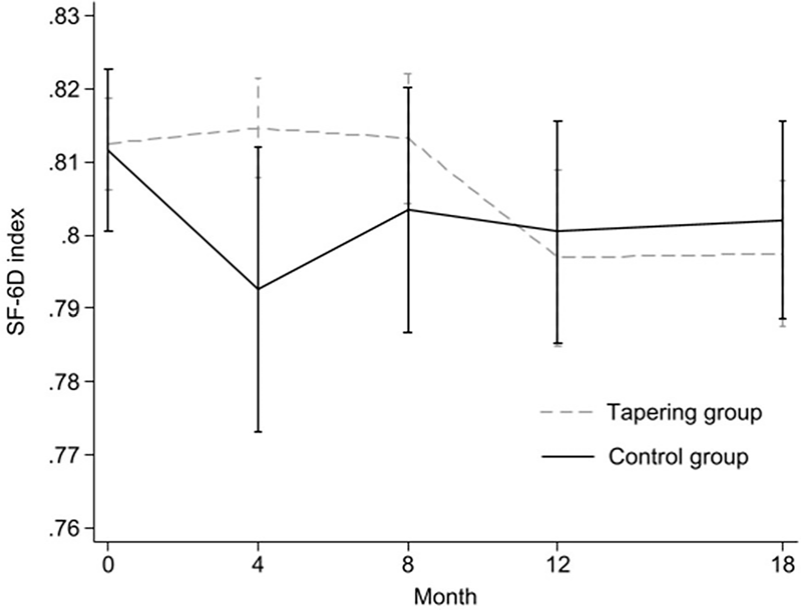
Mean SF-6D with 95% confidence interval during the study period. SF-6D, short form six-dimensional.

As presented in Figure 2, the mean SF-6D index was similar between groups throughout the study period. Mean 18-month QALYs were 1.21 (95% CI: 1.20-1.22) in the tapering group and 1.20 (95% CI: 1.19-1.22) in the control group, Table 2. The difference was statistically insignificant: 0.008 points (95% CI: −0.008 to 0.024). A sensitivity analysis with last observation carried forward if SF-6D values were missing did not alter the conclusion, mean between-groups difference: 0.005 (−0.011 to 0.022).

**Table 2:** QALY and rheumatology healthcare costs during the 18-month study period

Outcome	Tapering group *N* = 95	Control group *N* = 47	Difference between groups^a^ (95% CI)
	Mean per patient (95% CI)		
QALY	1.21 (1.20–1.22)	1.20 (1.19–1.22)	0.008 (-0.008–0.024)
Extra visits costs (€)	83 (63–102)	40 (3–76)	43 (2–84)
Number of extra visits due to symptoms of flare^b^	0.8 (0.61–0.99)	0.4 (0.03–0.73)	0.4 (0.02–0.81)
Biologic treatment costs (€)	3007 (2252–3763)	4987 (3321–6652)	−1979 (−3798 to −161)
Biological drug costs (€)	2698 (1951–3446)	4385 (2682–6088)	−1686 (−3536 to 163)
Cost due to switch of biological therapy (€)	15 (−6 to 35)	140 (−60 to 340)	−125 (−327 to 76)
Biologic administration costs (€)	294 (233–355)	462 (353–570)	−167 (−291 to −44)
Patients treated with intravenous biologics^c^	*N* = 42	*N* = 24	
Intravenous biologics costs (€)	581 (511–652)	789 (708–870)	−207 (−312 to −102)
Number of visits to receive intravenous infusions, *n*	8.4 (7.4–9.3)	11.3 (10.1–12.4)	−2.9 (−4.3 to −1.4)
Infusion seat time (hours)	16.6 (14.6–18.6)	22.5 (20.2–24.8)	−5.9 (−8.9 to −2.9)
Nurse time related to intravenous infusions (hours)	8.3 (7.3–9.3)	11.3 (10.1–12.4)	−3.0 (−4.5 to −1.5)
Patients treated with subcutaneous biologics^c^	*N* = 54	*N* = 24	
Subcutaneous biologics costs (€)	66 (54–77)	115 (79–152)	−50 (−87 to −12)
Number of visits to receive subcutaneous biologics	4.0 (3.6–4.4)	6.2 (5.7–6.7)	−2.2 (−2.8 to −1.5)
Nurse time related to subcutaneous biologics (hours)	1.1 (0.9–1.3)	1.9 (1.3–2.6)	−0.8 (−1.5 to −0.2)
Hospital costs (€)	3090 (2333–3847)	5026 (3363–6689)	−1936 (−3753 to −119)
Patient transport costs (€)	394 (272–516)	341 (221–460)	54 (−115 to 223)
Transport distance (km)^d^	839 (580–1098)	725 (469–980)	115 (−246 to 475)
Patient time costs (€)	528 (438–619)	634 (514–754)	−106 (−255 to 43)
Patient time costs (hours)	21.7 (18.0–25.4)	26.1 (21.1–31.0)	−4.4 (−10.5 to 1.8)
Time related to extra visits (hours)	0.6 (0.46–0.74)	0.3 (0.03–0.55)	0.3 (0.02–0.61)
Time related receiving biological treatment (hours)	8.0 (6.2–9.8)	12.8 (9.5–16.0)	−4.7 (−8.4 to −1.1)
Transport time (hours)	13.1 (10.3–15.9)	13.0 (9.6–16.5)	0.06 (−4.3 to 4.5)
Patient costs (€)	923 (720–1126)	975 (752–1197)	−52 (−350 to 246)

N, number; CI, confidence interval; QALYs, quality-adjusted life years; €, euro.

a Evaluated as tapering group-control group.

b Mean number of extra visits due to symptoms of flare per patient.

c One patient in the tapering group and 1 patient in the control group switched biological therapy due to persistent flare resulting in a switch between intravenous and subcutaneous administration.

d The distance from the individual patient’s home address to the hospital and back obtained from Google Maps.

### Hospital costs

#### Extra visit costs

During the 18-month study period, 54% (51/95) of the tapering group and 15% (7/40) of the control group had extra outpatient visits to evaluate possible symptoms of flare. Most patients required 1 extra visit (35% [33/95] vs 7% [3/47]), some 2 extra visits (14% [13/95] vs 4% [2/47]), and a few 3 or more extra visits (5% [5/95] vs 4% [2/47]). As presented in Table 2, the tapering group had statistically significantly more extra visits during the study period, between-group difference: 0.4 visit per patient (95% CI: 0.02-0.81). This resulted in a statistically significantly higher mean cost for extra visits in the tapering group, yielding a between-group difference of 43€ (95% CI: 2€-84€).

#### Biological treatment status

Six patients from the tapering group and 1 patient from the control group were lost to follow-up; missing data were imputed with their baseline biological dose after loss to follow-up. As reported previously, 75% (71/95) of the tapering group and 17% (8/47) of the control group were on a tapered biological dose at 18 months [6]. A ≥50% dose reduction compared with baseline was achieved in 37% (35/95) and 2% (1/47), respectively, while 15% (14/95) of the tapering group managed to discontinue their biological treatment.

As shown in earlier results, 32% (30/95) of patients in the tapering group and 2% (1/47) of patients in the control group reduced their biological dose by ≥50% and maintained LDA at month 18, thus achieved successful tapering [35]. The proportion of patients in the tapering group who reached successful tapering was similar for patients treated with TNFi (32% [28/88]) vs non-TNFi (29% [2/7]) [35]. However, when looking at patients in the tapering group treated with biosimilars (adalimumab, etanercept, or infliximab) vs originator biologics (abatacept, certolizumab-pegol, golimumab, and tocilizumab), a nonsignificantly higher proportion of patients treated with originator biologics reached successful tapering (28% [21/74] vs 43% [9/21], respectively). In the tapering group, rates of successful tapering for the individual biological drugs were 50% (4/8) for certolizumab-pegol, 50% (3/6) for golimumab, 41% (7/17) for adalimumab, 33% (2/6) for tocilizumab, 30% (6/20) for etanercept, 22% (8/37) for infliximab, and 0% (0/1) for abatacept. However, these results should be interpreted with caution as the numbers are very low and the trial is not powered for these analyses. Regarding potential predictors for achieving successful tapering, no statistically significant predictors have been identified in the BIODOPT trial despite evaluation of various baseline characteristics, including demographics, clinical-, and treatment-related outcomes [35,36].

#### Biological treatment costs

Switches of biological therapy due to persistent arthritis flare were required for 1 patient in the tapering group and 3 patients in the control group, whereas 1 patient in the tapering group switched biologics due to inflammatory bowel disease flare. Only 1 patient (from the control group) did not regain LDA after the switch. This patient was, due to persistent flare, switched 3 times during the study period before LDA was regained. Biological drug costs related to the switch were higher in the control group but did not reach statistical significance, mean group difference: −125€ (95% CI: −327€ to 76€), Table 2.

The tapering group had fewer visits in the outpatient clinic to receive biological treatments; between-group difference for intravenous biologics −2.9 visits per patient (95% CI: −4.3 to −1.4), and for subcutaneous biologics −2.2 visits per patient (95% CI: −2.8 to −1.5), Table 2. Consequently, the tapering group had a lower infusion set time to receive intravenous biologics and required less nurse time for biological administration, as presented in Table 2. This resulted in a statistically significantly lower mean cost for biological administration in the tapering group, between-group difference: −167€ (95% CI: −291€ to −44€).

The costs for biological drugs, ie, originators and biosimilars, were nonsignificantly lower in the tapering group than in the control group, mean group difference: −1686€ (95% CI: −3536€ to 163€). When looking only at biosimilar-treated patients (adalimumab, etanercept, or infliximab), mean costs in the tapering group were 1070€ (95% CI: 943€-1197€) compared with 1506€ (95% CI: 1288€-1725€) in the control group, data not shown. Thus, biosimilar-treated patients in the tapering group had statistically significantly lower biological costs than the control group: −437€ (95% CI: −687€ to −186€). When limiting the analysis to patients treated with nonbiosimilars, ie, originator biologics, costs for the biological products in the tapering group were 8438€ (95% CI: 6519€-10,356€) compared with 15,035€ (12,757€-17,312€) in the control group. This gave a markedly lower biological cost in the tapering group than in the control group: −6597€ (95% CI: −9418€ to −3777€).

The biological treatment cost was calculated by combining costs related to biological treatments, including the cost related to the biological switch to biologic administration. A statistically significantly lower biological treatment cost favouring the tapering group was demonstrated, mean group difference: −1979€ (95% CI: −3798€ to −161€). The between-group difference was still statistically significant when limiting the analysis to biosimilar-treated patients (difference: −804€ [95% CI: −1152€ to −456€]) as well as originator-treated patients (difference: −6613€ [−9429€ to −3797€]), data not shown.

#### Hospital costs

Hospital costs included biological treatment costs and extra visit costs. As presented in Table 2, hospital costs were statistically significantly lower in the tapering group than in the control group: −1936€ (95% CI: −3753€ to −119€). The between-group difference remained statistically significant when only assessing biosimilar-treated patients (difference: −772€ [95% CI: −1139€ to −404€]) as well as originator-treated patients (difference: −6530€ [−9340€ to −3720€]), data not shown.

**Figure 3:**
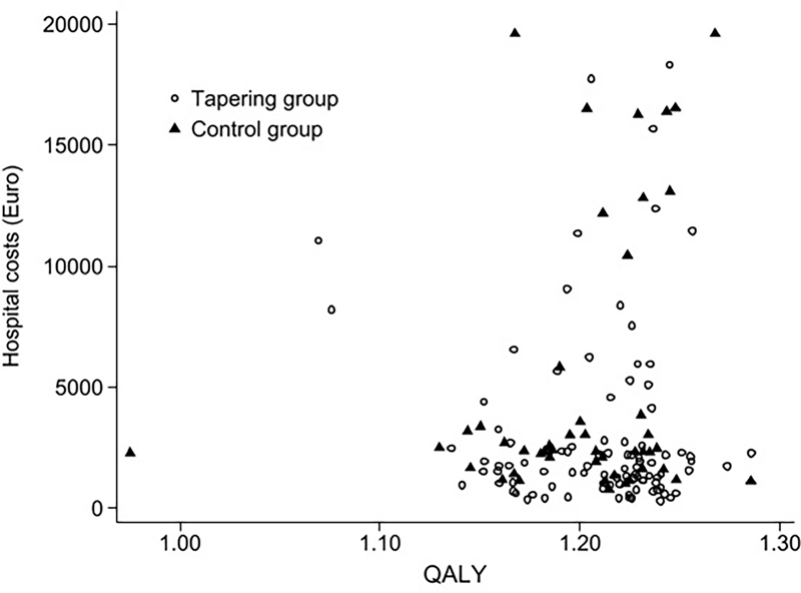
Scatterplot of individual hospital costs and QALY at 18 months. QALY, quality-adjusted life year.

Individual hospital costs and QALY are illustrated in Figure 3. No deterioration in QALY was observed in patients from the tapering group who had low hospital costs. Nor did patients from the control group with high hospital costs have higher QALY.

### Patient costs

#### Patient transport costs

The mean transport distance during the study period was 839 km (95% CI: 580-1098 km) in the tapering group and 725 km (95% CI: 469-980 km) in the control group, Table 2. Thus, no statistically significant between-group difference was observed in distance: 115 km (95% CI: −246 to 475 km) or transportation costs: 54€ (95% CI: −115€ to 223€).

#### Patient time costs

Mean transport time during the study period was similar, between-group difference: 0.06 hours (95% CI: −4.3 to 4.5 hours), Table 2. The tapering group spent more time on extra visits, mean group difference: 0.3 hours (95% CI: 0.02-0.61 hours), whereas the control group spent more time on receiving biological treatment, mean group difference: −4.7 hours (95% CI: −8.4 to −1.1 hours). When combining time related to transport, extra visits, and receiving biological treatment, no statistically significant between-group difference was observed: −4.4 hours (95% CI: −10.5 to 1.8 hours). Likewise, patient time costs did not reach statistical significance, mean group difference: −106€ (95% CI: −255€ to 43€).

#### Patient costs

Patient costs included patient transport costs and patient time costs. No statistically significant difference in patient costs was demonstrated, mean group difference: −52€ (95% CI: −350€ to 246€).

### Incremental cost-effectiveness ratio

The planned incremental cost-effectiveness ratio (ICER) was not calculated as the effect measure (ie, QALY) was similar between groups, and hospital costs favoured the tapering group. Thus, the tapering algorithm is evidently cost-saving while providing similar health outcomes in QALY.

### Subgroup analyses

Subgroup analyses on hospital costs in the tapering group stratified by LDA at month 18 did not reveal any statistically significant mean group difference: 413€ (95% CI: −1522€ to 2348€). When comparing patients in the tapering group treated with TNFi vs non-TNFi at baseline, a statistically significant lower cost for the TNFi treated was observed (as expected due to lower biosimilar costs): mean group difference −5772€ (95% CI: −10,318€ to −1226€). When stratifying by diagnosis, mean hospital costs in the tapering group were 3020€ (95% CI: 1822€-4217€) for patients with RA, 2183€ (95% CI: 1527€-2840€) for patients with PsA, and 3623€ (95% CI: 2146€-5101€) for patients with axSpA. Thus, a statistically significantly lower mean hospital cost was observed in the PsA tapering group than in the non-PsA tapering group (combined RA and axSpA tapering groups), *P* = 0.048 (data not shown). However, interpretation of these results must be made with caution as the number of patients with PsA allocated to tapering is small, and the trial was not powered for these subanalyses.

## DISCUSSION

To our knowledge, this is the first cost-effectiveness analysis evaluating disease activity–guided tapering to continuation of biologics as usual care with data on various biologics, including biosimilars. Moreover, the study is, as far as we know, the first to evaluate the cost-effectiveness of biological tapering in patients with PsA or axSpA. Disease activity–guided tapering of biologics, including biosimilars, was demonstrated to be cost-effective with a significant reduction in hospital costs while patient QALY and patient costs remained similar. When limiting the analysis to assess the impact of biosimilar drug prices, tapering of biological originators was proven to have a markedly higher cost-saving compared with when biosimilars were tapered. However, biosimilar tapering still resulted in a clinically relevant and statistically significant cost-saving, which emphasises that tapering remains relevant in the era of biosimilars.

Our results are in accordance with the majority of the current evidence in which originator biological tapering is found to be cost-effective as a substantial reduction in costs was demonstrated with no significant loss in QALY [[14], [15], [16]]. The randomised DRESS-RA trial evaluated disease activity–guided tapering of originator adalimumab and etanercept to continuation in patients with RA in sustained LDA [1]. The cost-effectiveness analyses demonstrated a large mean cost difference of 12,280€ (95% percentile: 10,502€ to 14,104€) per 18 months in favour of the tapering group [14]. Similar to our analysis, the DRESS-RA trial found no statistically significant difference in QALY (−0.02, 95% percentile: −0.07 to 0.02). Three-year data from the DRESS-RA trial showed that the tapering algorithm was still cost-effective [15].

The randomised STRASS trial assessed disease activity–guided tapering of originator adalimumab and etanercept to continuation in patients with RA in sustained remission [2]. The mean cost difference was 8440€ (95% CI: 6507€-10,212€) after 18 months with lower cost for the tapering group [17]. However, a small but statistically significant loss in QALY was observed in the tapering group, between-group difference 0.158 (95% CI: 0.085-0.232). Although the acceptability of QALY loss to cost savings was unclear, the tapering algorithm still seemed cost-effective.

Using a Markov model calibrated with data from DRESS-RA, STRASS, and the Nijmegen RA cohort, Verhoef et al [16] assessed 5 different tapering strategies for adalimumab and etanercept to continuation: 4-step tapering, 5-step tapering, tapering without withdrawal, use of strict flare criteria, and use of a theoretical predictor for successful tapering. The 4- and 5-step tapering strategies were more cost-effective, with cost savings of 7277€ and 7873€ and no significant deterioration in QALY. In 2 other studies using Markov models with a 30-year time horizon, van Esveld et al [37] and Birkner et al [38] evaluated 3 different biological tapering strategies in RA: 50% dose reduction (tapering), discontinuation, and 50% dose reduction followed by discontinuation (de-escalation). The 50% dose reduction (tapering) algorithm was more cost-effective in both studies, with the 30-year ICER at 115,157€ and 526,254€ per lost QALY. The substantial differences in ICERs were driven by variations in pharmaceutical costs due to the use of weighted averages and by declining biological costs due to expiring patents [37]. The ICERs were 74,226€ and 247,987€ per lost QALY for the de-escalation strategies and 67,137€ and 216,879€ per lost QALY for discontinuation, respectively. Thus, these data suggest that single-step 50% dose reduction, not disease activity–guided tapering, could be the optimal tapering algorithm long-term. Additional research is needed to assess this further.

Strengths of the BIODOPT trial include being investigator-initiated, no involvement from the pharmaceutical industry, successful implementation of the randomised design, few patients lost to follow-up, and minimal exclusion criteria, which enabled inclusion of a representative sample of real-life outpatients with inflammatory arthritis in treatment with originator and biosimilar biologics. Moreover, patient-related costs and real data from a randomised trial (instead of a simulation model) were included in this cost-effectiveness evaluation.

Some limitations to our study should be noted. The economic evaluation was not the primary objective of the BIODOPT trial; therefore, the cost-effectiveness analysis might be underpowered. Secondly, the study period was 18 months long, which only allows for 6 months follow-up after biologic withdrawal at 12 months (if possible). Thus, late flares requiring dose escalation could be missed in our analyses. However, the forthcoming 5-year data from BIODOPT will provide additional insights. Moreover, SF-6D was used for the QALY assessment instead of the more commonly applied European Quality of Life 5-Dimensions 5-Level index (EQ-5D-5L) as only the SF-36 questionnaire was collected in the study. Nonetheless, EQ-5D-3L could have been calculated from SF-36 with a mapping algorithm, but this method leads to information loss and increased uncertainty compared with collected EQ-5D data [39]. Previously, Lillegraven et al [40] showed diverging scores when comparing EQ-5D and SF-6D in patients with RA, particularly in patients with poor health and severe disability, ie, Health Assessment Questionnaires (HAQ) scores ≥2. We previously reported very low baseline HAQ scores (mean: 0.13 in both groups) with no significant between-group differences at 18 months [6]. In this paper, we demonstrated a mean SF-6D score of 0.82 in both trial groups at baseline, corresponding to good health. Based on the above, a potential difference in utility scores (ED-5D vs SF-6D) is expected to be minor and of less importance.

Due to the pragmatic design, some patients in the control group tapered their biological treatment, which can influence the cost-effectiveness results. However, this would lead to an underestimation of costs and possibly a slight deterioration in QALY; therefore, the current results are judged to be valid. Another limitation to consider is that the majority of patients were treated with TNFi, thus, limiting the generalisability to other biological treatments. Larger tapering trials with data on different biological modes of action are needed. Another aspect to address is that 3 different inflammatory arthritis diseases were included in this trial, which increases the risk of overlooking important differences between the diagnoses. Nonetheless, we found a significantly lower mean hospital cost among patients with PsA allocated to tapering compared with a combined group of patients with RA or axSpA, which potentially could indicate that patients with PsA were able to taper their biologics more aggressively. However, conclusions should be made with caution as the number of patients with PsA allocated to tapering is small, and the trial was not powered for the subanalysis. Future research on the cost-effectiveness of biological tapering, not only in PsA but also in axSpA, is encouraged, as current evidence is limited to patients with RA [[14], [15], [16], [17]].

Another aspect worth noticing is that loss of productivity, sick leave, and contacts to other health care professionals, such as general practitioners or physiotherapists, were not included in this cost-effectiveness analysis, as these data were not collected as part of the study. Some of this information could have been obtained through linkage to relevant Danish registers, but this would require a substantial additional workload. Meanwhile, as adverse event rates and persistent flare rates previously have been reported with no differences between groups [6], we did not anticipate a major difference in productivity loss, sick leave, or contacts to other health care professionals. Thus, we consider it unlikely that additional data obtained by linkage would significantly alter the overall conclusions.

Health care systems as well as treatment prices differ between countries. We used 2022 costs for the biological therapies in our analyses; thus, prices for biosimilar adalimumab, etanercept, and infliximab were applied. Although biosimilars may be used differently in other countries, the fact that our results demonstrate significantly lower hospital costs with the tapering algorithm provides important evidence that tapering is still relevant in the era of biosimilars. Future research on tapering with a long follow-up is needed as fewer flares are expected as time passes on continued tapered biological dosing; thus, over time, a larger cost-saving favouring the tapering strategy is expected.

## CONCLUSION

Disease activity–guided tapering of biological therapies in a biosimilar era resulted in a significant reduction in hospital costs, whereas the impact on QALY and patient costs was similar. Thus, the tapering algorithm was found to be cost-saving in comparison with usual care.

## CRediT authorship contribution statement

**Line Uhrenholt:** Writing – review & editing, Writing – original draft, Visualization, Validation, Software, Resources, Project administration, Methodology, Investigation, Funding acquisition, Formal analysis, Data curation. **Mads Engell Refstrup Sørensen:** Writing – review & editing, Visualization, Validation, Software, Project administration, Formal analysis, Data curation. **Jan Sørensen:** Writing – review & editing, Visualization, Validation, Supervision, Software, Resources, Methodology, Formal analysis, Conceptualization. **Anne Estrup Olesen:** Writing – review & editing, Validation, Supervision, Resources, Methodology, Conceptualization. **Robin Christensen:** Writing – review & editing, Supervision, Methodology, Conceptualization. **Lene Dreyer:** Writing – review & editing, Supervision, Resources, Methodology, Funding acquisition, Conceptualization. **Bente Glintborg:** Writing – review & editing, Supervision, Methodology. **Ellen-Margrethe Hauge:** Writing – review & editing, Supervision, Resources, Investigation. **Anne Gitte Loft:** Writing – review & editing, Supervision, Investigation. **Mads Nyhuus Bendix Rasch:** Writing – review & editing, Supervision, Investigation. **Hans Christian Horn:** Writing – review & editing, Supervision, Investigation. **Annette Schlemmer:** Writing – review & editing, Supervision, Funding acquisition. **Salome Kristensen:** Writing – review & editing, Visualization, Validation, Supervision, Resources, Project administration, Methodology, Investigation, Funding acquisition, Conceptualization.

## Competing interests

LU reports financial support was provided by The Danish Regions Medicine Grants, The Danish Rheumatism Association, The Health Science Research Fund of the North Denmark Region, Aase and Ejnar Danielsen Grant, Aalborg University Hospital, and Grant of Manufacturer Vilhelm Pedersen and Wife upon recommendation from the Novo Nordisk Foundation. Robin Christensen reports financial support was provided by The Oak Foundation. LD reports a relationship with Eli Lilly that includes speaking and lecture fees; Galderma that includes speaking and lecture fees; Janssen Pharmaceuticals, Inc that includes speaking and lecture fees; Bristol Myers Squibb Co that includes funding grants; and AbbVie Inc that includes funding grants. EMH reports a relationship with Novo Nordisk Inc that includes speaking and lecture fees and Novartis Pharmaceuticals Corporation that includes speaking and lecture fees. EMH reports a relationship with Independent Research Fund Denmark, Novo Nordic Foundation, Danish Rheumatism Association, Aarhus University, Danish Regions Medicine Grants, and Galapagos that include funding grants; AbbVie Inc that includes funding grants and travel reimbursement; Pfizer Inc that includes travel reimbursement; and Sobi Inc that includes travel reimbursement. AGL reports a relationship with Janssen Pharmaceuticals Inc that includes speaking and lecture fees; Novartis Pharmaceuticals Corporation that includes consulting or advisory and speaking and lecture fees; and Union Chimique Belge that includes consulting or advisory and speaking and lecture fees. MNBR reports a relationship with Sobi Inc that includes speaking and lecture fees. HCH reports a relationship with Janssen Pharmaceuticals Inc that includes consulting or advisory. BG reports a relationship with Sandoz Inc, AbbVie Inc, Bristol Myers Squibb Co, and The Danish Rheumatism Association that include funding grants. AS reports a relationship with Novartis Pharmaceuticals Corporation that includes speaking and lecture fees; Eli Lilly and Company that includes speaking and lecture fees; and Janssen-Cilag SAS that includes speaking and lecture fees. EMH has been the principal investigator in trials by SynACT Pharma and is involved as the site principal investigator in trials by AbbVie, Novartis, Novo Nordisk, and Sanofi. BG chairs the DANBIO steering committee. All other authors declare that they have no known competing financial interests or personal relationships that could have appeared to influence the work reported in this paper.
